# Food Modulation Controls Astaxanthin Accumulation in Eggs of the Sea Urchin *Arbacia lixula*

**DOI:** 10.3390/md16060186

**Published:** 2018-05-28

**Authors:** Christian Galasso, Ida Orefice, Alfonso Toscano, Tomás Vega Fernández, Luigi Musco, Christophe Brunet, Clementina Sansone, Paola Cirino

**Affiliations:** Stazione Zoologica Anton Dohrn di Napoli-Istituto Nazionale di Biologia, Ecologia e Biotecnologie Marine, Villa Comunale, 80121 Naples, Italy; christian.galasso@szn.it (C.G.); ida.orefice@szn.it (I.O.); alfonso.toscano@szn.it (A.T.); tomas.vegafernandez@szn.it (T.V.F.); luigi.musco@szn.it (L.M.); paola.cirino@szn.it (P.C.)

**Keywords:** food enrichment, aquaculture, nutraceutical, astaxanthin, *Arbacia lixula*

## Abstract

The carotenoid astaxanthin has strong antioxidant properties with beneficial effects for various degenerative diseases. This carotenoid is produced by some microalgae species when cultivated in particular conditions, and, interestingly, it is a predominant carotenoid in aquatic animals throughout a broad range of taxa. Recently, astaxanthin was detected in the eggs of the sea urchin *Arbacia lixula* in relevant concentrations when this organism was maintained in culture. These results have paved the way for deeper research into astaxanthin production by this species, particularly in regards to how astaxanthin production can be modulated by diet. Results showed that the highest content of astaxanthin in eggs was observed in sea urchins fed on a diet enriched with *Spirulina platensis*. This result was confirmed by the high antioxidant activity recorded in the egg extracts of these animals. Our results suggest that (i) the sea urchin *A. lixula* is able to synthesize astaxanthin from precursors obtained from food, and (ii) it is possible to modulate the astaxanthin accumulation in sea urchin eggs by modifying the proportions of different food ingredients provided in their diet. This study demonstrates the large potential of sea urchin cultivation for the eco-sustainable production of healthy supplements for nutraceutical applications.

## 1. Introduction

Marine photosynthetic organisms produce secondary metabolites that can be considered high-value bioproducts with beneficial effects for human health. Algae-derived products possess antioxidant properties, as well as anticancer and antimicrobial activities [1]. In particular, the red ketocarotenoid astaxanthin (3,3′-dihydroxy-carotene-4,4′-dione) is known as a highly biologically active molecule, and for this reason, it is significantly dominating the health care and aquaculture market. The high astaxanthin market request is due to its multiple known health benefits, from its pronounced antioxidant activity, to the most recent discovery of astaxanthin’s roles as an antidepressant, and in the prevention of UVA-induced skin photoaging [2,3]. Currently, commercial astaxanthin is mainly chemically synthesized, but it is also extracted from natural producers, such as the green algae *Haematococcus pluvialis*, the genetically mutated yeast *Xanthophyllomyces dendrorhous* (*Phaffia rhodozyma*), the bacteria *Paracoccus carotinifaciens* and *Lactobacillus* sp., and crustacean exoskeletons collected from shrimp processing waste [4,5]. However, among the aforementioned natural sources, only astaxanthin produced from *H. pluvialis* is accepted and guaranteed for human consumption. On the other hand, the common astaxanthin commercially available is produced by chemical synthesis using either the Wittig reaction or the Grignard reaction, and has recently been generating regulatory concern due to the potential toxic effects of the chemicals used for the synthesis [6].

The astaxanthin molecule has two identical chiral centers, and the synthetic astaxanthin exists in three configurational (stereo) isomers: (3*S*,3′*S*), (3*S*,3′*R*), and (3*R*,3′*R*). The natural astaxanthin synthesized by marine organisms is in the 3*S*, 3′*S* configuration, which is the only bioactive isomer [7]. Astaxanthin possesses strong scavenging activity against free radicals and other prooxidant molecules, protecting the lipid bilayer from peroxidation. This marked effect is due to its unique molecular structure (characterized by polar ionic rings and non-polar conjugated carbon–carbon bonds), which confers to astaxanthin an antioxidant property 10-fold greater than that of other carotenoids [3].

The production of astaxanthin from natural sources such as yeasts or algae has become one of the most successful activities in biotechnology, given its great demand in food, feed, nutraceutical, and pharmaceutical applications. The global astaxanthin market size was estimated at USD 555.4 million in 2016 [8].

Besides the practical advantages, by improving culture conditions and transformation processes supported by omics technologies, new sources and natural processes for the production of astaxanthin and other carotenoids are strongly desirable. Unfortunately, methodological (such as light induction and extraction techniques) or biotechnological issues (low production) are nowadays hindering the production of astaxanthin from biological sources [9].

Genetic engineering recently demonstrated that *Escherichia coli*, *Saccharomyces cerevisiae* and *Corynebacterium glutamicum* could be used as host strains for astaxanthin production by the introduction of genes involved in the biosynthesis pathway [5,10,11,12,13].

Beyond synthetic and natural production, there are some particular foods with a high content of astaxanthin. Increasing market growth regards sea urchin gonads, especially in Asia and North America, as high-quality food [14]. Garama and collaborators [14], analyzed dark colored gonads of *Evechinus chloroticus,* and found high amounts of fucoxanthin, fucoxanthinol, β-carotene, canthaxanthin, and astaxanthin. This HPLC analysis represents a helpful indication for the development of appropriate sea urchin diets, in order to enhance gonad color and relative carotenoid content, to increase the consumer perception and healthy properties of the final food product.

Another sea urchin, *Strongylocentrotus droebachiensis,* has been investigated for its potential as a new ingredient with beneficial effects [15]. In particular, the digestive tract and gonads of this species were analyzed for antioxidant activity, as well as phenol and flavonoid content. The results demonstrated that edible gonads could provide consumers with high biological value compounds, while digestive tracts might be used as an alternative source of antioxidants in food additives.

In a previous paper, Cirino et al. [16] detected high levels of bioactive astaxanthin in the eggs of the sea urchin, *Arbacia lixula*, fed with formulated food composed of animal (mussels) and plant (macroalgae, microalgae, and corn) ingredients. This last paper demonstrated that the farmed sea urchins contained higher concentrations of this active substance than wild ones. For this reason, here we manipulated the component proportions present in the Ration Blocks of Food (RBF) administered to farmed *A. lixula* individuals, in order to identify the ingredient mainly responsible for the astaxanthin bioconversion/accumulation in eggs. Moreover, we measured astaxanthin production in each experimental group of individuals fed with different formulations and assessed the bioactivity of astaxanthin extracted from their eggs.

The aim of the present study is to find the best food formulate that is able to enhance astaxanthin production in *A. lixula* eggs for an eco-friendly industrial application.

## 2. Results

### 2.1. Astaxanthin Concentration in Eggs

**Figure 1:**
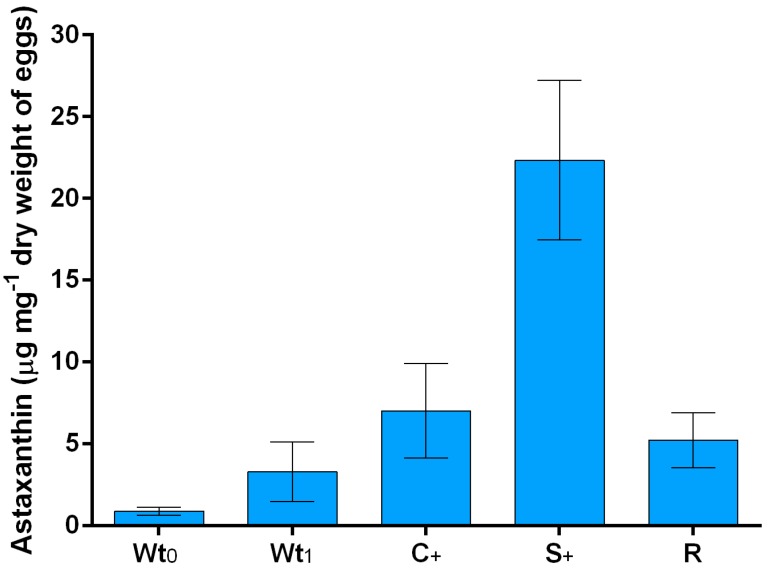
Astaxanthin in *Arbacia lixula* eggs. Concentrations were reported as µg mg^−1^ of dry weight of egg for: Wild individuals harvested from the field at the beginning of the experiment (Wt_0_); wild individuals harvested from the field at the end of the experiment maintained without feeding (Wt_1_); farmed individuals fed with a diet enriched with corn (C_+_); farmed individuals fed with a diet enriched with *Spirulina platensis* (S_+_) and control group fed with the Ration Blocks of Food (R).

Astaxanthin was the main pigment in the eggs of *Arbacia lixula*, with concentrations ranging between 0.89 and 22.33 μg mg^−1^ of egg dry biomass (Figure 1).

Astaxanthin concentration was determined in wild *A. lixula* eggs at two experimental times: soon after the animals were collected (at time 0; Wt_0_), and after 7 weeks, where wild animals were maintained in tanks without feeding (Wt_1_). These two group of animals constituted our control, that is, organisms not influenced by our feed modulation.

The astaxanthin content in wild *A. lixula* collected at the beginning (Wt_0_) and at the end of the experiment (Wt_1_) did not increase during the study period (0.89 ± 0.25 μg mg^−1^ vs. 3.30 ± 1.82 μg mg^−1^; Table 1, Wt_1_ vs. Wt_0_ and Figure 1). The astaxanthin concentration significantly increased in reared *A. lixula* when compared to the wild sea urchins (Table 1, Wt_1_ vs. R). Moreover, individuals fed with a diet enriched with *Spirulina platensis* (S_+_) showed a huge increase in the concentration of astaxanthin compared to the control group (R) fed with Ration Blocks of Food (RBF) (22.33 ± 4.89 μg mg^−1^ vs. 5.23 ± 1.68 μg mg^−1^; Table 1, S_+_ vs. R and Figure 1). Sea urchins fed with RBF enriched in corn (C_+_) content did not show a significant improvement in astaxanthin production (7.02 ± 2.88 μg mg^−1^, Table 1, C_+_ vs. R and Figure 1). Interestingly, astaxanthin was not detected in food samples, suggesting the transformation of other carotenoids or their precursors into astaxanthin in sea urchins.

### 2.2. Radical Scavenging Activity

Radical scavenging ability was tested on radical species 2,2-DiPhenyl-1-PicrylHydrazyl (DPPH) of the *A. lixula* eggs’ methanol extracts, after feeding the animals with different diets (R, C_+_, S_+_) for 7 weeks. It was also tested on wild individuals at the beginning (Wt_0_) and at the end (Wt_1_) of the experiment. In particular, the most active group was that of the animals fed with RBF enriched in *S. platensis* content (S_+_), showing a strong dose-dependent reduction of the purple radical DPPH into the yellow reduced form (36.9%, 78.5%, and 94.7%, at 0.1, 1, and 10 μg mL^−1^ respectively, Table 2). Extracts from every sea urchin group showed some radical scavenging activity at any concentration tested.

## 3. Discussion

Natural astaxanthin is considered to be a “super antioxidant” for its important applications in the nutraceuticals, cosmetics, food, and aquaculture industries. Astaxanthin can significantly reduce free radicals and oxidative stress, thereby enhancing health. Astaxanthin is a predominant carotenoid in marine animals and has strong quenching and scavenging effects against free radicals. For this reason, astaxanthin has been defined as a “super vitamin E” [17]. With the ongoing increase in demand, astaxanthin is one of the most high-value marine-derived products of the future [3,18,19].

Generally, carotenoids present in animal tissues are directly accumulated from food, or partly modified through metabolic reactions, reflecting the diet of the animals [20]. Carotenoids are used for pigmentation in aquaculture and ornamental fish. Synthetic and natural astaxanthin from *Phaffia* yeast and *Haematococcus* algae is widely used for the pigmentation of salmon, trout, and red sea bream. Astaxanthin is safe, accumulating in tissues after being ingested with no side effects [21].

Astaxanthin originating from phyto- and zooplankton components in the diet is naturally accumulated in the integuments and gonads of many marine animals through the food web. Among others, this carotenoid was found in some jellyfish and corals, crustaceans, mollusks, and echinoderms [20]. In some echinoderms, such as sea urchins, the carotenoid echinenone was found [22]. In our study, the accumulation of astaxanthin in the eggs of reared sea urchins seems arguably to be the result of its de novo synthesis through a hypothetical biotransformation pathway from astaxanthin precursors, such as carotenoids present in the diet. Astaxanthin is produced from β-carotene [23], which is present in almost all algae, together with other bioactive pigments [24,25]. Following the carotenoid biosynthetic pathway in plants [23], the two main enzymatic pathways from β-carotene are the biosynthetic route through β-cryptoxanthin and zeaxanthin, or through echinone and canthaxanthin. Zeaxanthin is present in cyanophytes (such as *S. platensis*) and green algae, in which it acts as a photoprotectant. *S. platensis* is known to contain carotenoids such as zeaxanthin, β-cryptoxanthin, lutein, β-carotene, and echinone [26]. The presence of these carotenoids in *S. platensis*, many of them acting as potential precursors of astaxanthin, could explain the high astaxanthin concentration found in the eggs of *A. lixula* reared on a diet enriched with *S. platensis*. In line with Martin et al. [23], the present study supports the hypothesis that *A. lixula* is able to transform astaxanthin precursors like β-carotene, zeaxanthin-cryptoxanthin, and echinon-canthaxanthin into astaxanthin through metabolic processes.

By modulating ingredients in the food formulation, we here optimized astaxanthin production, thereby providing astaxanthin precursors within the RBF consumed by sea urchins.

The importance of these findings can be exemplified as follows. On the one hand, astaxanthin concentration increases by 750% (22 ± 4.8 µg mg^−1^ of S_+_ vs. 2.6 ± 1.8 µg mg^−1^ of Wt_1_ of dry weight of eggs) when sea urchins are fed with a diet enriched with *S. platensis,* in comparison to wild sea urchins. Hence, the astaxanthin content in reared sea urchins fed with *S. platensis* reaches ≈3700 µg·individual^−1^ compared to 143 µg·individual^−1^ in wild sea urchins, which represents about a 26-fold enrichment. It means that using one reared individual is equivalent, in terms of the astaxanthin provided, to using 26 wild sea urchins. In practice, it would be enough to process two reared sea urchins fed with *Spirulina platensis*, instead of 49 wild sea urchins, to cope with the daily need for astaxanthin of an adult human (recommended dose of ≈7 mg [27]).

Further investigations need to be conducted in order to identify the molecular pathway involved in the metabolic production of astaxanthin in *A. lixula*. The identification of the molecular pathway involved in astaxanthin production by *A. lixula* will suggest the best food formulation for supplying carotenoid precursors able to upregulate the gene involved.

## 4. Materials and Methods

### 4.1. Culture System

*Arbacia lixula* individuals ranging from 37.3–56.3 mm (test diameter) were hand collected by scuba-diving from the rocky shores of the Gulf of Naples, along the southern Tyrrhenian coast of Italy. The animals were placed in a cooler and carried to the laboratory under moist conditions within 2 h.

Sampled sea urchins designated for the study were held in suspended baskets in a semi-closed recirculating system that received very low flows of make-up seawater for water losses associated with routine tank cleaning. Water temperature was 16 ± 1 °C throughout the experiment. Aeration in the tanks provided additional water movement and air supply. Water quality parameters were set to maintain a healthy recirculation system [28].

### 4.2. Feeding Practice

We produced different diets by varying the original recipe of Ration Blocks of Food (RBF) described in Cirino et al. [16]. Prior to the start of the experiment, three groups of 15 individuals each were randomly sorted from the wild sea urchins collected at t_0_ (45 animals taken from Wt_0_ group). The groups of 15 individuals were maintained in separate baskets in the same tank and fed with different diets, as detailed below.

We formulated three different RBF diets, differing from each other in the relative quantities of the ingredients, namely macroalgae (*Ulva lactuca)*, blue-greens (*S. platensis*), corn, mussel meal, fish oil, and mineral supplement. The different RBF types are detailed in Table 3.

RBFs were produced by weighing and mixing ingredients according to the feeding practice described in Cirino et al. [16]. The finished ration blocks, weighing on average 1 g, were stored at −20 °C, and were used to feed the sea urchins twice a week for 7 weeks before the collection of gametes.

### 4.3. Collection of Gametes

Gametes were collected by manually pouring from the gonadal pores after animal sacrifice. This method allowed us to gather pure fresh gametes. Only eggs were selected for successive analysis. After collection, they were immediately frozen in liquid nitrogen and stored at −80 °C.

### 4.4. Experimental Design and Statistical Analysis

The effect of different diets on the concentration of astaxanthin in *A. lixula* was tested by feeding the sea urchins with several different diets. A total number of 75 sea urchins were allocated in five experimental groups. These were farmed sea urchin individuals fed with a diet enriched with corn (C_+_); reared individuals fed with augmented *S. platensis* content (S_+_); wild individuals harvested from nature at the beginning of the experiment (Wt_0_); wild animals harvested from the field at the end of the experiment (Wt_1_); and a control group fed with the standard ration block (R).

The effect of different diets on the astaxanthin concentration per unit of dry egg mass (AC, in µg mg^−1^) was tested as the effect of a fixed factor named Experimental group, fixed, with five levels: C_+_; S_+_; Wt_0_; Wt_1_; and R. A test of the sea urchins’ diameter (in mm) was introduced in the model as a covariate in order to extract potential variability due to differences in the size of the individuals [29]. An ANCOVA was computed on the root-transformed AC after checking the homogeneity of variances via Levene’s test. A set of four planned comparisons were defined a priori in order to test for the relevant hypotheses [30] and coded as contrast vectors (L_i_) according to Neter et al. [31]. The vectors coding for testing the effect of diet enrichment were L_1_ (C_+_ vs. R) and L_2_ (S_+_ vs. R). L_3_ coded the test for differences between wild and farm conditions (Wt_1_ vs. R). An additional comparison was added to control for natural variation of astaxanthin concentration in time, coded by L_4_ (Wt_1_ vs. Wt_0_).

### 4.5. Chemical Extraction from Eggs

Extraction methodology was in accordance with Sansone et al. [25] and Cutignano et al. [32]. The extraction procedure was conducted under dark conditions, at room temperature, and under nitrogen atmosphere in order to avoid oxidation of the sample. Freeze-dried eggs were extracted with 1 mL methanol for 15 min, then the mixture was sonicated and centrifuged at 5000 rpm for 10 min at 12 °C. The supernatant was transferred to a clean tube. The methanol extracts were dried in a rotary vacuum evaporator (Buchi rotavapor R-114, Flawil, Switzerland). The dry extract was stored under nitrogen atmosphere at −20 °C prior to analysis.

### 4.6. HPLC Analysis

Pigment analysis was conducted by High Performance Liquid Chromatography (HPLC) on an aliquot of the aqueous methanol extract (22–185 mg of dry egg), according to the method described by Orefice et al. [33]. Briefly, the extract was injected in a reversed-phase column (C8 Kinetex column; 50 mm × 4.6 mm; 2.6 µm particle size, Phenomenex^®^, Wilmington, NC, USA) of the Hewlett Packard series 1100 HPLC (Hewlett Packard, Wilmington, NC, USA). Pigments were detected by diode-array spectroscopy (spectrum data collected in the range of 350–750 nm) using a Hewlett Packard photodiode array detector, model DAD series 1100, and absorbance chromatogram was reported at 440 nm. The identification and quantification of pigments was carried out using pigment standards from the D.H.I. Water & Environment (Horsholm, Denmark) [34].

### 4.7. DPPH-Assay

The 2,2-DiPhenyl-1-PicrylHydrazyl (DPPH) was used for the radical scavenging assay (Sigma Aldrich, Saint Louis, MO, USA, cat. 257621). Various concentrations (0.1, 1, and 10 µg mL^−1^) of egg extract were mixed with a final concentration of DPPH of 0.1 mM in methanol, allowed to react for 30 min in the dark, and absorbance was measured at 517 nm using a microplate reader. Results were presented as a percentage of DPPH reduction with respect to methanol negative control and α-tocopherol used as the positive control.

## 5. Conclusions

Our study stresses the fact that the cultivation of *Arbacia lixula* with controlled feeding strongly enhances the astaxanthin content in the sea urchins’ eggs. This result paves the way for the technological use of *A. lixula* in biological astaxanthin production.

*A. lixula* cultivation can contribute to the expansion of the nutraceutical market, providing a new source of a highly sought-after natural supplement, with widely recognized antioxidant activity. Moreover, gonads coming from wild animals can present an accumulation of harmful contaminants, such as arsenic, cadmium, chromium, nickel and lead [35,36], which constitute a high risk for consumers. For this reason, aquaculture seems to be a valid alternative for the production of highly valuable food, with higher safety for consumers.

This perspective also opens up the possibility of the development of unconventional aquaculture of non-edible species, with strong biotechnological potential.

## Figures and Tables

**Table 1:** Analysis of covariance on root-transformed astaxanthin concentration per unit of dry egg mass (μg mg^−1^). Diameter of sea urchin test (mm) was the covariate and the effect of diet was tested across experimental groups. Contrast vectors (L_i_) coded for planned comparisons between pairs of experimental groups specified between brackets. Experimental groups comprised wild individuals harvested from the field at the beginning of the experiment (Wt_0_); wild individuals harvested from the field at the end of the experiment (Wt_1_); farmed individuals fed with a diet enriched with corn (C_+_); farmed individuals fed with a diet enriched with *S. platensis* (S_+_) and control group fed with the Ration Blocks of Food (R). SS stands for Sum of Squares, d.f. for degrees of freedom, MS for Mean Squares, F is the Fisher’s statistic, and *p* is the observed level of significance.

Source of Variation	SS	d.f.	MS	F	*p*
Intercept	<0.01	1	<0.01	<0.01	0.9636
Diameter	0.18	1	0.18	2.54	0.1276
Experimental group	2.45	4	0.61	8.71	**0.0004**
L_1_: (C_+_ vs. R)	0.08	1	0.08	1.14	0.3000
L_2_: (S_+_ vs. R)	0.34	1	0.34	4.87	**0.0399**
L_3_: (Wt_1_ vs. R)	0.38	1	0.38	5.40	**0.0313**
L_4_: (Wt_1_ vs. Wt_0_)	0.01	1	0.01	0.08	0.7832
Error	1.34	19	0.07		

**Table 2:** Radical scavenging capacity (Inhibition percentage, IP, %) of methanol extract of *Arbacia lixula* eggs on DPPH free radicals. Values reported are a percentage of the activity of extracts versus a blank and are represented as the mean ± standard deviation (SD) of five independent samples. Readings were performed three times to check accuracy.

	Concentration Tested		
	0.1 μg mL^−1^	1 μg mL^−1^	10 μg mL^−1^
**Sea urchin eggs**			
Wt_0_	15.6 ± 13.9	22.0 ± 11.2	32.6 ± 24.9
Wt_1_	14.1 ± 3.5	26.1 ± 18.3	33.0 ± 1.9
C₊	22.8 ± 12.4	61.3 ± 9.9	75.7 ± 21.7
S₊	36.9 ± 13.9	78.5 ± 3.1	94.7 ± 19.5
R	15.4 ± 4.3	75.0 ± 14.2	86.3 ± 7.5
**Food Formulations**			
RBF S₊	7.9 ± 2.6	3.9 ± 3.25	4.2 ± 0.56
RBF C₊	7.9 ± 1.25	7.6 ± 1.78	13.3 ± 0.9
RBF R	14.5 ± 1.5	6.1 ± 3.5	1.5 ± 1.45

**Table 3:** Description of different food formulations (RBF) for feeding of the experimental sea urchin groups in culture.

Group	Food Items and Formulations
RBF-R	Standard Ration Blocks of Food with mussels, macroalgae (*U. lactuca*), *S. platensis* and corn
RBF-S_+_	Ration Blocks of Food with mussels and macroalgae (*U. lactuca*), reinforced in *S. platensis* and deprived of corn
RBF-C_+_	Ration Blocks of Food with mussels and macroalgae (*U. lactuca*), reinforced in corn content and deprived of *S. platensis*
